# A critical look at the practice and culture of science with calls to action

**DOI:** 10.1038/s42004-023-00855-z

**Published:** 2023-03-20

**Authors:** Hind A. Al-Abadleh

**Affiliations:** Department of Chemistry and Biochemistry, Wilfrid Laurier University, Waterloo, ON N2L 3C5 Canada

**Keywords:** Chemical education, Communicating chemistry

## Abstract

Efforts are ongoing to address inequities in scientific fields. Here, the author provides a critical look at the practice and culture of science with calls to action to broaden participation and recognition of talented members from marginalized groups in the chemical sciences.

As chemists, we have crucial roles to play in guiding humanity’s transition to a socially peaceful, just, and more sustainable future in the era of climate change through research, education, community engagement, and evidence-based policies. Our journey starts with concerted and conscious efforts to make transformative changes to the ‘scientific culture’ that hinders the full participation and advancement of talented members from marginalized groups in the chemical sciences. This paper is the second excerpt from my lecture for the inaugural Canadian Society of Chemistry (CSC) Gilead Award for Excellence in Equity, Diversity, and Inclusion. The first excerpt of the award lecture was recently published^1^. As an environmental chemist who started her academic career in 2005 in Canada at Wilfrid Laurier University, I highlight here my analysis and reflections of the culture of science and end with calls to action.

The last two years have shaken the consciousness of human societies because of the COVID-19 global pandemic, extreme weather events, fatal incidences of racial injustices, discoveries of mass graves of Indigenous children in Canada, refugee crises, and ongoing armed conflicts. Also, the last two years have shown the world how scientists work collaboratively and in real time to develop new vaccines, design new tools and technologies to detect and analyze airborne pathogens, purify indoor air, and quantify improvements to outdoor air quality because of lockdowns. We also witnessed unprecedented levels of openness to talk publicly about uncomfortable truths pertaining to equity, diversity, and inclusion (EDI) in the sciences, including chemistry. The ongoing practice and culture of science has failed marginalized communities and mostly being used to cement the power of oppressors partly leading to systematic social and economic inequities and racism^2^. Leaders in academic institutions, scientific societies, funding agencies, and publishers have finally acknowledged the need to shift the root causes of inequities facing marginalized groups from individuals to institutional cultures and policies that historically enabled the exclusion of the ‘other’.

## Doing Science: ingredients and culture

**Fig. 1:**
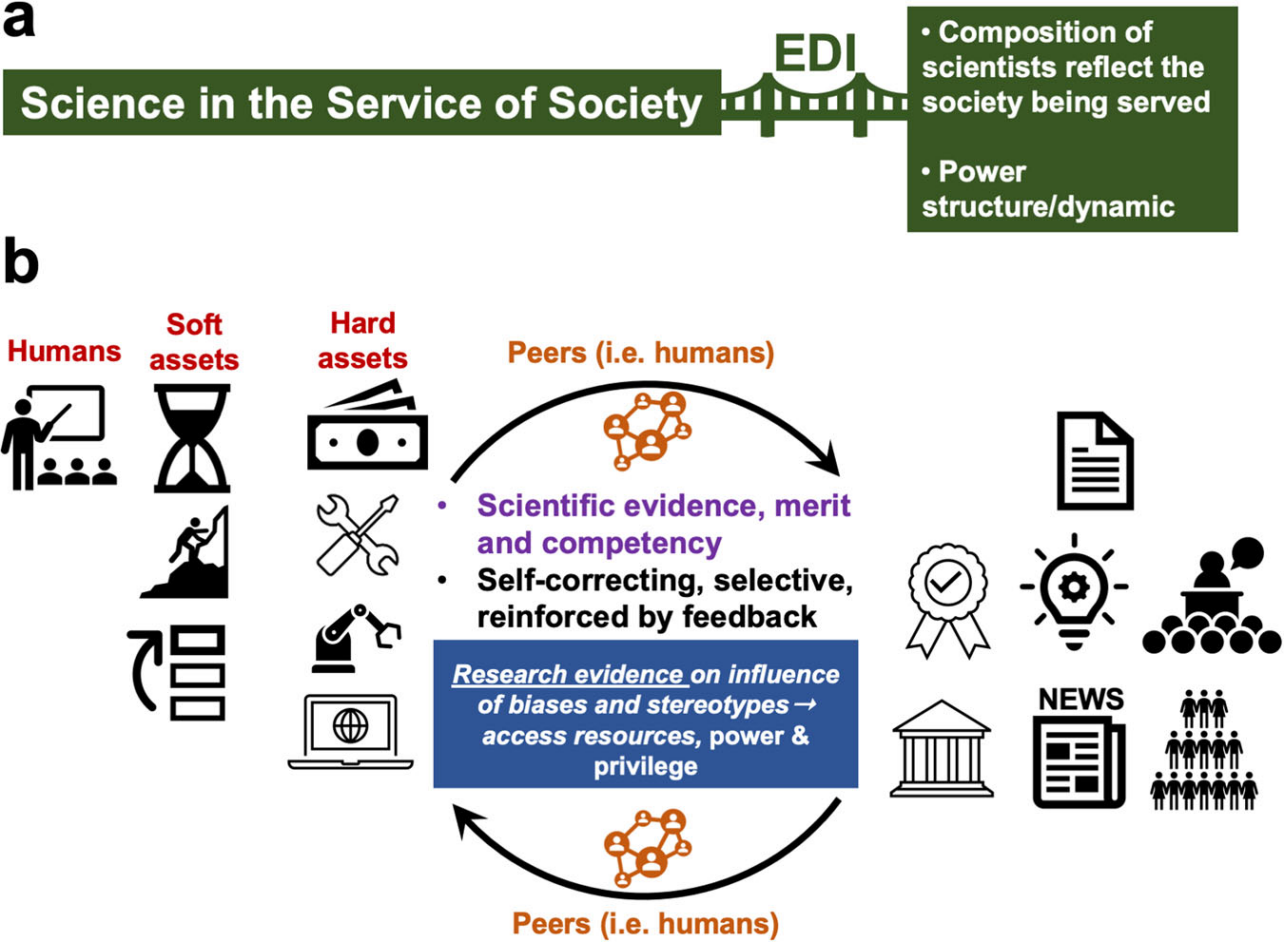
Illustration of the scientific culture. The top section (**a**) shows that EDI efforts aim to bridge the gap between practicing science to serve society and the composition of scientists and power structure/dynamics within scientific fields. The lower section (**b**) highlights the ingredients for doing science, which include humans, soft, and hard assets (left side). The right side highlights the different forms of scholarly outputs and recognitions. The middle section highlights the role of peers in evaluating the scientific evidence, merit and competency of scientists, and the prized notion that science is self-correcting, selective, and reinforced by feedback. See text for details.

Figure 1 shows an illustration of the ongoing practice and culture of science. EDI efforts bridge the gap between the higher objective of doing science, which is to be in the service of society, and the composition/diversity/power structure and dynamics within the scientistic community. The composition of the scientific community should -ideally- reflect the society being served and its changing demographics. However, composition alone is not enough as it does not equal power. Even in “diverse” settings, scientists from under-represented groups continue to be marginalized in scientific fields^3^. The ingredients for doing science are: (a) humans, who include mentors and students^4^. The pool from which scientists come from is the general population of a given country or community, (b) soft assets, which include time, freedom of thought, persistence, ability to prioritize, hard work, ability to bounce back, good daily habits, and (c) hard assets, which include money, space, tools, instruments, and computers. The combination of these ingredients enables knowledge generation through original research, i.e., scientific scholarly output, which comes in the form of peer-reviewed papers, new products, new policies, or changes to existing ones. Some of that scholarly output has short- and/or long-term benefits to the wider communities locally and globally.

The peers in each scientific field are also humans, who use the scientific evidence presented in the scholarly output to judge and rank the merit of ideas and competency of other scientists. Peers also judge and rank the quality of the scholarly output and stature of other scientists to justify recommendations for more, or less, assets needed to do the science. Recognitions of this scholarly output come in the forms of invitations to speak at conferences, invitations to join expert panels, awards, promotions and more titles, and coverage by science journalists in the media (TV, radio, newspapers, etc.). Therefore, the practice of science is labeled as self-correcting, selective, and reinforced by feedback from peers leading to the illusion among scientists that doing science is the most objective human endeavor for the pursuit of knowledge with little room for subjectivity. However, as detailed in the next section, while scientists aspire to be as objective as they can, they are not immune to historical and ongoing social struggles that manifest in the form of racism, sexism, ableism, and phobias of all kind leading to propagating an image about the scientific community as an exclusive club for elites.

## Influence of biases and stereotypes

There is ample research evidence^5–10^ that shows the influence of biases and stereotypes in the peer-review process that limits or hinders accessing power and privilege (and hence resources), influences a culture driven by interests, and affects the choice of words used in describing scholarly output and excellence of marginalized researchers. Hence, this research evidence clearly shows that the practice of science takes place on uneven-playing fields because (a) there is competition for limited resources that is more Darwinian^11,12^ than transparent, fair, and ethical, (b) there is complex inter- and intra-research group dynamics fueled by individual worldviews, career aspirations, lived experiences, and professional networks. These group dynamics are a reflection of the rigged education system^3,13^ and white supremacy in science that enable systems of oppression and racialization in science^14^, and (c) there are senior scientists in each scientific field who play a major role in the making of future field leaders through the advancement and retention of a selected few that they deem worthy of their time, energy, and backing. Dr. Malinda Smith, Vice-Provost and Associate Vice-President Research (EDI) at the University of Calgary and co-authors, wrote a chapter in the book entitled The Equity Myth: Racialization and Indigeneity at Canadian Universities on the twelve unconscious race and gender biases in the academy that they refer to as The Dirty Dozen listed in Table 1^15^. This list was compiled based on the evidence published in social and political sciences journals.

**Table 1:** A side-by-side list of twelve persistent biases in academic institutions^15^ and principles to reduce bias and discrimination in science, technology, engineering, and mathematics (STEM)^17^.

Persistent biases in academic institutions^15^	Actionable principles to reduce bias and discrimination in STEM^17^
What happens before? Biases before graduate school	Learn the basics
Gifted or helpful? Biases in letters of reference	Acknowledge your biases and your privilege
Who speaks? Biases in classrooms	Do your research, listen to your friends and colleagues, and then be vocal
Who presents? Biases in conferences	Be strategic about who you work with
What counts? Citational and self-promotion biases	Restructure retention and advancement programs
Whom do you know? Academic networks and social capital	Generate a code of conduct as a team
What must you know? Canonical and curriculum biases	Be inclusive
Do you fit? Affinity bias and homosocial reproduction	Be intentional
Where are you from? Résumé racism and accent bias	Be supportive
Bossy or brilliant? Biases in teaching evaluations	Rethink the status quo in science
Where’s the help? Service, structural ingratitude, and the girl scouts tax	Make action a habit
Who leads? Separate and unequal career tournaments	Embrace the final thoughts that “Science is done by (some) people and for the benefit of (some) people”, “small changes can have a big effect”, and that “Every one of us has the power to make a difference” in changing the culture in STEM

## Calls to action

To level the playing fields, we need to go beyond equity to justice, where scientists in powerful positions of authority and influence fix the systems to offer equitable access to tools, opportunities and recognition and not put the onus of the change in the culture of science on the marginalized^16^. Dr. Smith and co-authors^15^ offer directions for policy changes and evidence-based practices to address their research findings and encourage universities to “evaluate and monitor their goals, priorities, and practices through an equity lens.” This is because, “today’s universities need strong leadership that will disrupt organizational incrementalism and, in the process, facilitate change,” and “institutions must provide space for new scholars, accepting that their new and additional forms of knowledge, ideas, interests, and cultural networks will enhance the scholarship, pedagogy, and community relationships within the university.” To reduce bias and discrimination in STEM, Mills et al.^17^ outlined twelve actionable principles listed in Table 1 that trainees, principal investigators, departments and faculties can use to affect change. In addition, bold and ambitious statements backed by action plans were released in the past few years from several organizations such as the Chemical Institute of Canada through their Working towards Inclusion, Diversity, and Equity committee^18^, the Natural Sciences and Engineering Research Council of Canada through their Dimensions Charter^19^, Canada Research Chairs through their EDI requirements and practices^20^, research publications^21^ by the Royal Society of Chemistry^22^, the American Chemical Society^22,23^, Nature portfolio^22^, Science magazine^22,24^, and De Gruyter^22^ to name a few, which acknowledged that EDI work needs to be done at the editorial board level and throughout the peer review process. Almost all academic, government, and private sector institutions created administrative positions for EDI officers, managers, and Vice Presidents to revise and create new policies that address EDI challenges. I wish to highlight here what Springer Nature’s *Communications Chemistry* did to diversify the expertise and composition of their editorial board as a new open access journal in the Nature Portfolio family of journals, which I recently joined as a member of the editorial board: they created a Google form^25^ for anyone interested in joining the editorial board with a list of questions. Then, the Chief Editor follows up and conducts virtual interviews with candidates followed by their decision on moving forward.

Lastly, to affect institutional EDI in a world facing changing demographics and climate change, we—as members of the chemistry community—must acknowledge the centrality of human diversity to advancement and innovation in chemical research, education, and outreach to wider communities. Our goal should be to capitalize on the potential and talent of traditionally marginalized groups in chemistry by putting in place accountable and effective systems, policies, and programs that address the root causes of inequities head-on, welcome, encourage, support, retain, and advance the contributions of all. This way, we level the playing scientific fields and create a culture that acknowledges the spectrum of strengths brought to the table by individuals at all education and career stages without aiming to conform them to a template familiar to the majority. Also, this way, we—as chemists—will pride ourselves for working in a field that has the potential to guide humanity’s transition to a better future for people and the planet.
